# Firing activities of a fractional-order FitzHugh-Rinzel bursting neuron model and its coupled dynamics

**DOI:** 10.1038/s41598-019-52061-4

**Published:** 2019-10-31

**Authors:** Argha Mondal, Sanjeev Kumar Sharma, Ranjit Kumar Upadhyay, Arnab Mondal

**Affiliations:** 1 Computational Neuroscience Center, University of Washington, Seattle, Washington USA; 2 Department of Mathematics & Computing, Indian Institute of Technology (Indian School of Mines), Dhanbad, 826004 India

**Keywords:** Applied mathematics, Computational science

## Abstract

Fractional-order dynamics of excitable systems can be physically described as a memory dependent phenomenon. It can produce diverse and fascinating oscillatory patterns for certain types of neuron models. To address these characteristics, we consider a nonlinear fast-slow FitzHugh-Rinzel (FH-R) model that exhibits elliptic bursting at a fixed set of parameters with a constant input current. The generalization of this classical order model provides a wide range of neuronal responses (regular spiking, fast-spiking, bursting, mixed-mode oscillations, etc.) in understanding the single neuron dynamics. So far, it is not completely understood to what extent the fractional-order dynamics may redesign the firing properties of excitable systems. We investigate how the classical order system changes its complex dynamics and how the bursting changes to different oscillations with stability and bifurcation analysis depending on the fractional exponent (0 < *α* ≤ 1). This occurs due to the memory trace of the fractional-order dynamics. The firing frequency of the fractional-order FH-R model is less than the classical order model, although the first spike latency exists there. Further, we investigate the responses of coupled FH-R neurons with small coupling strengths that synchronize at specific fractional-orders. The interesting dynamical characteristics suggest various neurocomputational features that can be induced in this fractional-order system which enriches the functional neuronal mechanisms.

## Introduction

Collective oscillatory dynamics and synchronous activity are the fundamental phenomena in dynamical systems^1,2^. It has both theoretical importance and biophysical significance in computational neuroscience. Mathematical biophysical models^3–6^ are the primary tools to characterize the nervous system. The foremost exciting step in neural dynamics is to understand the system architecture of individual neurons in terms of mathematical models of membrane potential. Various types of spiking and bursting are the dynamical responses of excitable cells^7,8^. Such type of research analyzes the chaotic behavior of excitable systems. When mathematical models are described as a single neuron or network, then the nonlinear dynamical techniques are applied to study the emerging oscillatory patterns and the synchronization phenomena. The classical order dynamical models depend on the immediate previous response, however the fractional-order derivative depends on all the previous responses, so it has a memory effect^9–11^. It can produce a different kinds of multiple time scale neuronal dynamics^12^. It^13–16^ provides a wide range in understanding the rich dynamical and neuronal responses. Fractional-order calculus originated from a letter written to Leibnitz by L’Hospital^13–15^. Now, it has become a promising and reliable mathematical tool that includes hereditary properties or memory dependence phenomena^13–17^. The discussion of multiple time scale dynamics has been studied in some previous articles^18,19^ which have the potential importance in signal processing. It has been studied that the firing rate of neocortical pyramidal neurons with the injected applied sinusoidal current can be well approximated with fractional-order derivative^20^.

Many researchers have worked on the fractional-order dynamical systems^21,22^. Results show that it follows power-law dynamics^23–25^. In human memory, the power-law dynamics was investigated earlier^26,27^, the accuracy of memory dependence decays at a rate nearly equal to *t*^−*α*^ where $0 < \alpha  < 1$. The power-law adaptation helps in describing some dynamical behavior of biological systems^12,28^. In recent years, fractional-order derivative has become very useful in modeling biological phenomena^13–16,29^, viscoelastic properties of tissue^30^, tissue electrode interface^31^, the kinetic property of drug delivery^32,33^, diffusion process^34–36^, biophysical neuron models and neural networks^37–42^. It has been found that cognitive behaviour can be modelled using fractional dynamics^43^. It was observed that fractional-order dynamics is used in vestibular oculomotor system^44^ and fly motion of sensitive neurons H1^28^. It can include the mechanism of synapses^28^ and the geometrical properties of excitable cells^44,45^. Single neuron models are analyzed by using fractional-order dynamics such as Hodgkin-Huxley (H-H), Morris-Lecar (M-L), FitzHugh-Nagumo (FHN), Hindmarsh-Rose (H-R) models, etc^10,39,46–49^. In this study, it has been demonstrated that the fractional-order dynamics of a solitary nerve membrane can be analyzed by using a suitable biophysical model that exhibits elliptic bursting. It reflects the rate of change of information through membrane voltage that leads to previous history-dependent activities. Different parameter regimes corresponding to qualitatively different dynamical properties are analyzed. This article investigates the FH-R neuron^50–52^ in a fractional-order domain to get a general idea about the spike patterns and spike frequency with stability and bifurcation scenarios. The relation between spiking and bursting is a significant question as well as a fascinating phenomenon in mathematical neuroscience, especially in neural coding. Bursting presents a recurrent transition between repetitive spiking and quiescent state. The switching phases depend on the strength of the slowly changing current stimulus to the dendrite. An exciting feature of the elliptic bursting is that the frequency of emerging spiking activity and ceasing the spiking is nonzero; at that time, the amplitude of the oscillations may be small^53^. It was experimentally studied that this type of bursting can be found in trifacial nerves controlling the jaw movement of rodents^54^.

It has been previously found that the slow variable in the 2D FHN model creates mathematical complexity that allows various dynamics for the membrane voltage of the neuron model including chaos. Therefore, fractional-order FH-R single neuron model has an excellent qualitative feature that exhibits many diverse oscillations of action potentials. Necessary and sufficient conditions are investigated for asymptotic stability analysis of the fractional-order commensurate FH-R model. Bifurcation shows the qualitative changes between the quiescent state and the oscillatory state^5,6^. The fractional-order FH-R model is investigated with a certain fixed-parameter sets, and extreme numerical computations are derived for examining the dynamical characteristics with analytical analysis using the fractional exponent as a predominant parameter. As a consequence, this generalization of the classical order model can produce biophysical variability. We present the effect of the fractional-order dynamics on the synchronization criterion in different ensembles for coupled oscillators. We observed different dynamical behavior with various fractional-orders that were not present in the classical order model.

## Fractional-Order FH-R Model

FitzHugh and Rinzel introduced FH-R model (1976, in an unpublished article)^50,52,53,55^, which is the modification of the classical FHN neuron model. The 2D FHN model^3,4^ illustrates a geometrical explanation of interesting biophysical phenomena that are relevant to neuronal excitabilities and spike generation. It exhibits continuous spiking with a specific external stimulus. However, it is not capable of generating various fascinating firing patterns produced in cortical neurons. FH-R neuron model which is the improved version of the FHN model, can produce abundant firing activities for some parameters when it is varied in a specific fixed range. The fast-slow subsystems describe the model; the fast subsystem consists of classical FHN equation^3^. The slow subsystem is one dimensional. It is biologically plausible and computationally efficient single neuron model. The commensurate fractional-order FH-R model is described as

1 $$\begin{array}{rcl}\frac{{d}^{\alpha }v}{d{t}^{\alpha }} & = & v-{v}^{3}/3-w+y+I={f}_{1}(v,w,y),\\ \frac{{d}^{\alpha }w}{d{t}^{\alpha }} & = & \delta (a+v-bw)={f}_{2}(v,w,y),\\ \frac{{d}^{\alpha }y}{d{t}^{\alpha }} & = & \mu (c-v-dy)={f}_{3}(v,w,y),\end{array}$$

where *v*, *w* and *y* represent the membrane voltage, recovery variable and slow modulation of the current respectively. *I* measures the constant magnitude of external stimulus current, and *α* is the fractional exponent which ranges in the interval $(0 < \alpha \le 1)$. *a*, *b*, *c*, *d*, *δ* and *μ* are the system parameters. The system reduces to the original classical order system when $\alpha =1$. *μ* indicates a small parameter that determines the pace of the slow system variable, *y*. The fast subsystem (*v*-*w*) presents a relaxation oscillator in the phase plane where *δ* is a small parameter. *v* is expressed in mV (millivolt) scale. Time *t* is in ms (millisecond) scale^4,6,11^. It exhibits tonic spiking or quiescent state depending on the parameter sets for a fixed value of *I*. The parameter *a* in the 2D FHN model corresponds to the parameter *c* of the FH-R neuron model^53,55^. If we decrease the value of *a*, it causes longer intervals between two burstings, however there exists a relatively fixed time of bursting duration. With the increasing of *a*, the interburst intervals become shorter and periodic bursting changes to tonic spiking.

The relation between injected current stimulus and membrane potential to generate an action potential, i.e., spike^10^, was previously introduced. The ideal resistor-capacitor theory describes the passive cell membrane dynamical analysis and the non-ideal resistor-capacitor circuit diagrams can characterize the oscillatory behavior^10,16,39,56,57^. The theory preserves the membrane voltage behavior. It plays a significant role in analyze the dielectric behavior of the cell membranes^16,39,57^. It was observed in experimental results that fractional-order dynamics follow a general power-law relation^10,56^. In the electrical activities of neurons, it was shown that the power-law dynamics follows $\alpha =0.76$ and 0.86 for warm and cold frog sciatic neurons respectively^39^. The non-ideal capacitor theory for current-voltage relation was described by a fractional-order derivative as follows, $C\frac{{d}^{\alpha }V}{d{t}^{\alpha }}=I$ where $0 < \alpha  < 1$. It follows a power-law dynamics and preserves the memory effects in the variations of the membrane voltage^9–11,16,39^. We consider this contrast in the fractional-order condition. The membrane voltages and specific membrane potential changes may instigate seizure-like activity in epilepsy^55^. It may cause reactions in the muscles for the specific strength of the stimulus. This type of bursting phenomenon can be explored in a more general way so that it may span in different research areas^55^. Let us study the fractional-order fast-slow system that contributes different firing activities which appears and disappears with the change of fractional-order exponents at the various set of predefined fixed parameters.

## Method

### Numerical solution scheme

To examine the fractional dynamics of FH-R model, we consider the most familiar definition of the fractional derivative in Caputo sense^13–15^. Consider the fractional-order derivative of a variable x(*t*) for the fractional exponent $\alpha \in (0,1)$ as folllows

2 $$\frac{{d}^{\alpha }{\rm{x}}}{d{t}^{\alpha }}=f({\rm{x}},t),$$

using the definition, we have

3 $$\frac{{d}^{\alpha }{\rm{x}}}{d{t}^{\alpha }}=\frac{1}{\Gamma (1-\alpha )}\,{\int }_{0}^{t}\,{(t-\tau )}^{-\alpha }{\rm{x}}^{\prime} (\tau )d\tau ,$$

where gamma function is defined as $\Gamma (z)={\int }_{0}^{\infty }\,{e}^{-u}{u}^{z-1}du$. An additional advantage of Caputo order derivative is that the derivative of a constant is zero. It is efficient to integrate all the previous activities of the function weighted by a function that follows power-law dynamics. Now, applying the L1 scheme^9,41,42,58^ on Eq. (3), approximating the fractional-order derivative as

4 $$\frac{{d}^{\alpha }{\rm{x}}}{d{t}^{\alpha }}\approx \frac{{(dt)}^{-\alpha }}{\Gamma (2-\alpha )}[\mathop{\sum }\limits_{k=0}^{N-1}\,[{\rm{x}}({t}_{k+1})-{\rm{x}}({t}_{k})]\,[{(N-k)}^{(1-\alpha )}-{(N-1-k)}^{(1-\alpha )}]],$$

and combining Eqs (2) and (4), the numerical solution of Eq. (2) can be formulated as

5 $$\begin{array}{rcl}{\rm{x}}({t}_{N}) & \approx  & {(dt)}^{\alpha }\Gamma (2-\alpha )f({\rm{x}},t)+{\rm{x}}({t}_{N-1})\\  &  & -\,[\mathop{\sum }\limits_{k=0}^{N-2}\,[{\rm{x}}({t}_{k+1})-{\rm{x}}({t}_{k})]\,[{(N-k)}^{(1-\alpha )}-{(N-1-k)}^{(1-\alpha )}]],\end{array}$$

where, *t*_*k*_ represents the *k*^*th*^ time step and ${t}_{k}=k\Delta t$. The variable x is considered as ${\rm{x}}\equiv (v,w,y)$ in our numerical results. Approximation of the fractional-order derivative for the membrane voltage (*v*(*t*)) is given by

6 $$\begin{array}{rcl}v({t}_{N}) & \approx  & {(dt)}^{\alpha }\Gamma (2-\alpha ){f}_{1}({\rm{x}},t)+v({t}_{N-1})\\  &  & -\,[\mathop{\sum }\limits_{k=0}^{N-2}\,[v({t}_{k+1})-v({t}_{k})]\,[{(N-k)}^{(1-\alpha )}-{(N-1-k)}^{(1-\alpha )}]].\end{array}$$

Similarly, we can derive numerically the expressions for other two variables (*w* and *y*) of Eq. (1). Hence the numerical solution of Eq. (2) can be summarized as the difference between the markov term weighted by the gamma function and the memory trace. Memory trace has the main functional role in the fractional-order system as it integrates all the past activities. The markov term weighted by the gamma function is given by ${(dt)}^{\alpha }\Gamma (2-\alpha )f({\rm{x}},t)+{\rm{x}}({t}_{N-1})$ and the memory trace is given by $[\mathop{\sum }\limits_{k=0}^{N-2}\,[{\rm{x}}({t}_{k+1})-{\rm{x}}({t}_{k})]\,[{(N-k)}^{(1-\alpha )}-{(N-1-k)}^{(1-\alpha )}]]$. The memory trace has no effect for $\alpha =1$ and the fractional-order system behaves like classical order model. The nonlinearity in the memory trace increases as we decrease the fractional-order *α* from 1 and the system dynamics depends on time. The fractional-order FH-R system is numerically integrated by using this scheme. We have considered different sets of parameters as follows^53,55^ $a=0.7,\,b=0.8,\,d=1,\,\delta =0.08$, $c=-\,0.775$ and $\mu =0.0001$, set I: $I=0.3125$, set II: $I=0.4$, set III: $\mu =0.18$, $I=3$, set IV: $c=1.3$, $\mu =0.0001$ and $I=0.3125,$ set V: $c=-\,0.908$, $\mu =0.002$ and $I=0.3125$ and remaining parameters are similar as above. We perform the analysis of FH-R model with these parameter sets. The system shows different firing patterns like elliptic bursting, tonic spiking/regular spiking, fast-spiking and high amplitude single spike with small amplitude oscillations. The different firing activities together with the mode transitions are investigated for different parameter regimes corresponding to qualitatively various dynamical behavior of a nerve cell.

### The characteristics of the fractional-order biophysical model

#### Stability analysis

The fixed points of the system (1) are derived as ${w}^{\ast }=({v}^{\ast }+a)/b$, ${y}^{\ast }=(c-{v}^{\ast })/d$ and ${v}^{\ast 3}-3{v}^{\ast }p=q$, where $p=(1-\frac{1}{b}-\frac{1}{d})$ and $q=(3I-\frac{3a}{b}+\frac{3c}{d})$ respectively. Depending on the nature of the discriminant of the cubic polynomial $F({v}^{\ast })={v}^{\ast 3}-3{v}^{\ast }p=q$, the system (1) can have maximum three equilibrium states. Throughout this study, the assumption (A) $bd < d+b$ holds (based on the numerical data).

##### **Proposition I**.

The cubic function *F*(v*) is strictly increasing and there exists only one branch of equilibrium state $E(q)=({v}^{\ast }(q),\frac{{v}^{\ast }(q)+a}{b},\frac{c-{v}^{\ast }(q)}{d})$, with $q\in {\mathbb{R}}$, for system (1), where ${v}^{\ast }(q)={F}^{-1}(q)$.

**Proof:** We have $F({v}^{\ast })={v}^{\ast 3}-3{v}^{\ast }p$ and $F^{\prime} ({v}^{\ast })=3{v}^{\ast 2}-3p$. The discriminant of *F*′ is given by $D(F^{\prime} )=\frac{36}{bd}(bd-d-b)$. Using the assumption (A), we obtain $D(F^{\prime} ) < 0$ that implies $F^{\prime} ({v}^{\ast }) > 0$ and the function *F* is strictly increasing (and invertible) on ${\mathbb{R}}$. Thus, it has only one real root ${v}^{\ast }(q)={F}^{-1}(q)$. The Jacobian of the system (1) at the fixed point $E({v}^{\ast },{w}^{\ast },{y}^{\ast })$ is given by

$$J({v}^{\ast })=(\begin{array}{ccc}1-{v}^{\ast 2} & -1 & 1\\ \delta  & -\delta b & 0\\ -\mu  & 0 & -\mu d\end{array}).$$

The characteristic polynomial is

$$\begin{array}{rcl}Q(\lambda ) & = & {\lambda }^{3}-(1-{v}^{\ast 2}-\delta b-\mu d){\lambda }^{2}\\  &  & +\,(\delta -\delta b+\mu -d\mu +bd\mu +b\delta {v}^{\ast 2}+d\mu {v}^{\ast 2})\lambda \\  &  & -\,(bd\delta \mu -b\delta \mu -d\delta \mu -bd\delta \mu {v}^{\ast 2}).\end{array}$$

From assumption (A) and ${\rm{\det }}\,(J)=\mu \delta ((bd-b-d)-bd{v}^{\ast 2}) < 0$, we obtain that at least one of the roots of the characteristic polynomial *Q*(*λ*) is negative. Considering the value of the parameter $d=1$ (which is constant and fixed for all the parameter sets), we have $Q(-\mu )=\mu (b\delta -\mu )$. If $\mu  < b\delta$ then $Q(-\mu ) > 0$, which implies that at least one real root of *Q*(*λ*) lies in $(-\infty ,-\,\mu )$ otherwise the root lies in $[\,-\,\mu ,0)$. We will discuss the case when $\mu  < b\delta$ for analytical treatment and proceeding in the similar way, we can also derive the analytical results for the case when $\mu  > b\delta$.

The system changes its stability through Hopf bifurcation and it occurs when the trace of the Jacobian matrix vanishes i.e., $1-{v}_{H}^{2}-\delta b-\mu d=0$, which gives ${v}_{H}=-\,\sqrt{1-\delta b-\mu }$ (say *γ*_1_) and ${v}_{H}=\sqrt{1-\delta b-\mu }$ (say *γ*_2_). *v*_*H*_ denotes the system variable where Hopf bifurcation occurs.

##### **Proposition II**.

The equilibrium state *E*(*q*) of system (1) is asymptotically stable (independent of the fractional exponent, *α*) for any $q\le F({\gamma }_{1})$ or $q\ge F({\gamma }_{2})$.

**Proof:** Suppose if we take the situation where $q\le F({\gamma }_{1})$, then ${v}^{\ast }={v}^{\ast }(q)={F}^{-1}(q)\le {\gamma }_{1} < 0$. Also if $q\ge F({\gamma }_{2})$, then ${v}^{\ast }={v}^{\ast }(q)={F}^{-1}(q)\ge {\gamma }_{2}$. It can be obtained that in both the cases $Q(1-{v}^{\ast 2}-\delta b-\mu ) < 0$. Thus, the negative real root (say *λ*_1_) of *Q*(*λ*) lies in $(1-{v}^{\ast 2}-\delta b-\mu ,-\,\mu )$ and other two roots satisfy ${\lambda }_{2}+{\lambda }_{3}=1-{v}^{\ast 2}-\delta b-\mu -{\lambda }_{1} < 0$ and ${\lambda }_{2}{\lambda }_{3}=\frac{{\rm{\det }}(J)}{{\lambda }_{1}} > 0$ respectively. From the above discussion, we can conclude that the roots lie on the negative real axis, so the equilibrium state *E*(*q*) is asymptotically stable and independent of the fractional exponent.

##### **Proposition III**.

If $q\in (F({\gamma }_{1}),F({\gamma }_{2}))$, then the equilibrium state *E*(*q*) of system (1) is asymptotically stable iff $(1-{v}^{\ast 2}-\delta b-\mu -{\lambda }_{1})\sqrt{-{\lambda }_{1}} < 2\sqrt{-det(J)}\,\cos \,\frac{\alpha \pi }{2}$, or equivalently,

7 $$\alpha  < \frac{2}{\pi }\arccos (min(1,\,max(0,\frac{(1-{v}^{\ast 2}-\delta b-\mu -{\lambda }_{1})\sqrt{-{\lambda }_{1}}}{2\sqrt{-det(J)}}))),$$

where ${\lambda }_{1}={\lambda }_{1}(q)\in (-\infty ,-\,\mu )$ is the smallest root of the characteristic polynomial *Q*(*λ*).

**Proof:** We have already shown that the smallest root ${\lambda }_{1}={\lambda }_{1}(q)\in (-\infty ,-\,\mu )$ and the other two roots of *Q*(*λ*) satisfy ${\lambda }_{2}+{\lambda }_{3}=1-{v}^{\ast 2}-\delta b-\mu -{\lambda }_{1}$, ${\lambda }_{2}{\lambda }_{3}=\frac{{\rm{\det }}(J)}{{\lambda }_{1}} > 0$. Now, the roots *λ*_2_ and *λ*_3_ satisfy the asymptotic stability condition $|{\rm{\arg }}(\lambda )| > \frac{\alpha \pi }{2}$ iff ${\lambda }_{2}{\lambda }_{3} > 0$ and $\frac{{\lambda }_{2}+{\lambda }_{3}}{\sqrt{{\lambda }_{2}{\lambda }_{3}}} < 2\,\cos (\frac{\alpha \pi }{2})$ hold^59^. Substituting the values of ${\lambda }_{2}+{\lambda }_{3}$ and *λ*_2_*λ*_3_, we obtain the condition (7).

#### Numerical results

Assumption (A) holds for all the above mentioned sets of parameters, hence there exists only one branch of equilibrium state *E*(*q*) for the system (1). We can also find ${\gamma }_{1}=-\,\sqrt{1-0.8\,\ast \,0.08-0.0001}=-\,0.9674$, ${\gamma }_{2}=\sqrt{1-0.8\,\ast \,0.08-0.0001}=0.9674$ and $F(-0.9674)=-\,4.5331$, $F(0.9674)=4.5331$ for parameter sets I and II respectively. From the proposition II, *E*(*q*) is asymptotically stable for any $q\le F({\gamma }_{1})$ or $q\ge F({\gamma }_{2})$, or equivalently, for any $I < 0.1390$ or $I > 3.1610$, independent of *α*. When $I\in (0.1390,3.1610)$, the equilibrium state *E*(*q*) becomes unstable for some values of *I* and *α*, and the stability criteria is given by the proposition III for this range. Now, suppose the value of applied stimulus $I=0.3125\in (0.1390,3.1610)$, the equilibrium point is stable for all $\alpha  < 0.80828$ (set I). Similarly, if we choose $I=0.4$, the equilibrium point is stable for $\alpha  < 0.6951$ (set II). The condition $\mu  > b\delta$ holds for the parameter set III and the stability of the equilibrium solution is given by the same condition (7). Thus, the equilibrium point is asymptotically stable for all $\alpha  < 0.95665$. The equilibrium point for the set IV is $E(0.54648,1.5581,0.75352)$ and the system (1) has the real eigen values given by *λ*_1_, *λ*_2_ and ${\lambda }_{3}=(\,-\,0.00028055,0.0613089,0.576231)$ respectively. Therefore, the equilibrium point *E* is a saddle point. Further, the equilibrium point is stable for $\alpha  < 0.956455$ at parameter set V.

The chaotic behavior of the system (1) can be obtained for the different parameter sets using the necessary condition $\alpha  > (2/\pi )\,{\tan }^{-1}\,(|{\rm{Im}}(\lambda )|/{\rm{Re}}(\lambda ))$^60^. We consider the parameter set I. The system has one real equilibrium point $E(-0.885098,-\,0.231373,0.110098)$ and the eigenvalues at this equilibrium point is given by *λ*_1_, *λ*_2_ and ${\lambda }_{3}=(\,-\,0.000196427,0.076349\pm 0.245811i)$. The equilibrium point E is a saddle point with index 2^60,61^. Now, using the above condition we obtain that the system (1) exhibits chaos for $\alpha  > 0.80828$. At the parameter set II, the equilibrium point and the eigenvalues are $E(\,-\,0.841243,-\,0.176554,0.066243)$ and *λ*_1_, *λ*_2_ and ${\lambda }_{3}=(\,-\,0.000204006,0.114207\pm 0.219938i)$ respectively. Here, the equilibrium point *E* is a saddle point with index 2. In this case, the system exhibits chaos for $\alpha  > 0.6951$. Proceeding in the similar way, the system exhibits chaos for $\alpha  > 0.95665$ and $\alpha  > 0.956455$ for the parameter sets III and V respectively.

#### Bifurcation analysis

**Figure 1:**
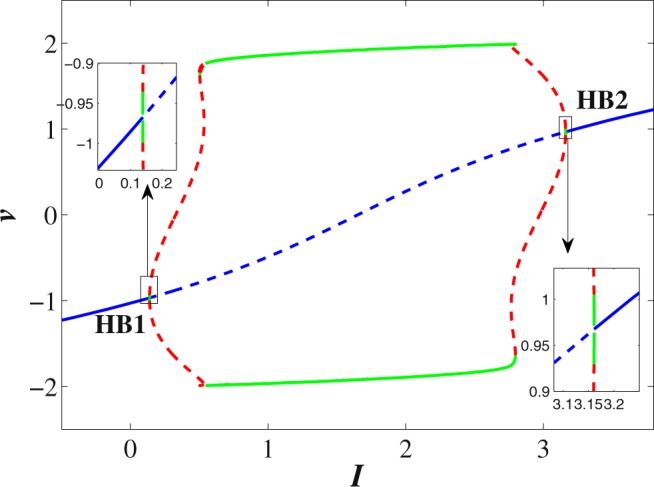
The bifurcation scenario of a classical order FH-R model with respect to the parameter *I* and keeping other parameters at their base values. The thick solid and the dotted blue lines indicate the stable and unstable steady states of the system respectively. Green and the dotted red lines indicate the stable and unstable limit cycles respectively. HB1 and HB2 represent the Hopf bifurcation points.

**Figure 2:**
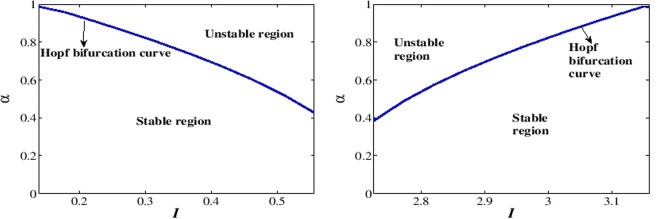
The bifurcation results of the fractional-order system (1) are plotted for parameter sets I and II respectively.

The bifurcation analysis for the classical order FH-R model is performed using the MATCONT software. The applied stimulus (*I*) is treated as the predominant parameter and other parameters are fixed at their base values with $\mu =0.0001$. The quiescent state disappears through super-critical Hopf bifurcations at $I=0.138716$ (HB1) and $I=3.161277$ (HB2) respectively (see Fig. 1). The thick blue line in the figure indicates the stable equilibrium state while the dotted blue line indicates the unstable equilibrium state. The system has stable focus node for $I < 0.138716$ and $I > 3.161277$ respectively. The system has saddle focus for $0.13872 < I\le 0.6$ and $2.6 < I\le 3.16128$ respectively. The FH-R model shows elliptic bursting at $I=0.3125$ (set I)^53^, however as we increase the value of *I* ($0.4 < I\le 0.6$), it shows co-existence of regular spiking with bursting. Further, with the increase of *I*, the system exhibits regular spiking. The system has saddle point for $0.6 < I\le 2.6$ and in this region the system first shows regular spiking and then it shows the first spike latency with the increase of *I*. For $2.6 < I\le 3.16128$, the system first shows the regular spiking with first spike latency, however with the increase of *I*, it shows the irregular bursting with first spike latency. The thick green line indicates the stable limit cycle whereas the red dotted line indicates the unstable limit cycle. In the classical order model, Andronov-Hopf bifurcation indicates the local birth and death of periodic solution whereas in the case of fractional-order model the nearby solutions of Hopf bifurcation are considered as the solution of the system. The stability of the fractional-order system around any equilibrium point is characterized by the sign of the variable ${n}_{i}(\alpha ,I)=\frac{\alpha \pi }{2}-|{\rm{\arg }}({\lambda }_{i}(I))|$, $i=1,2,3$. The system is said to be stable or unstable around the equilibrium point if ${n}_{i}(\alpha ,I) < 0$ or ${n}_{i}(\alpha ,I) > 0$. The variable ${n}_{i}(\alpha ,I)$ has similar role as the real part of the eigenvalue in the classical order system. Therefore, the condition for the occurrence of Hopf bifurcation in fractional-order system can be stated as^62^: (I) The Jacobian matrix *J* has two complex-conjugate eigenvalues and one real eigenvalue i.e., $D(Q({I}^{\ast })) < 0$, where I* is the critical value of the predominant parameter, (II) ${n}_{i}(\alpha ,{I}^{\ast })=0$ and ${\lambda }_{3}({I}^{\ast })\ne 0$, (III) ${\frac{\partial n}{\partial I}|}_{I={I}^{\ast }}\ne 0$. The Hopf bifurcation occurs in the system (1) when the parameter $I\in (0.1390,3.1610)$. Figure 2(a,b) show the stable/unstable region in the $(I,\alpha )$ - plane for equilibrium state *E*(*q*) with parameter sets I and II respectively. The blue curve in the figure refers to the Hopf bifurcation curve for the critical values α* given by the equation obtained from ${n}_{i}(\alpha ,{I}^{\ast })=0$. Suppose, we take $I=0.3125$, then the critical value is ${\alpha }^{\ast }(0.3125)=0.80828$. The system (1) is asymptotically stable when $\alpha  < {\alpha }^{\ast }(0.3125)$ and unstable for $\alpha  > {\alpha }^{\ast }(0.3125)$. Hopf bifurcation exists in the sysetm (1) for $\alpha ={\alpha }^{\ast }(0.3125)$. We have not considered the region $I\in [0.7,2.6]$ for the numerical analysis as $D(Q(I)) > 0$. The equilibrium state is asymptotically stable for most of the values of the fractional exponent *α* and unstable for very few values of *α*. For $I=0.4$, the critical value for the Hopf bifucation is ${\alpha }^{\ast }(0.4)=0.6951$.

#### Excitatory responses of the single fractional-order FH-R model

**Figure 3:**
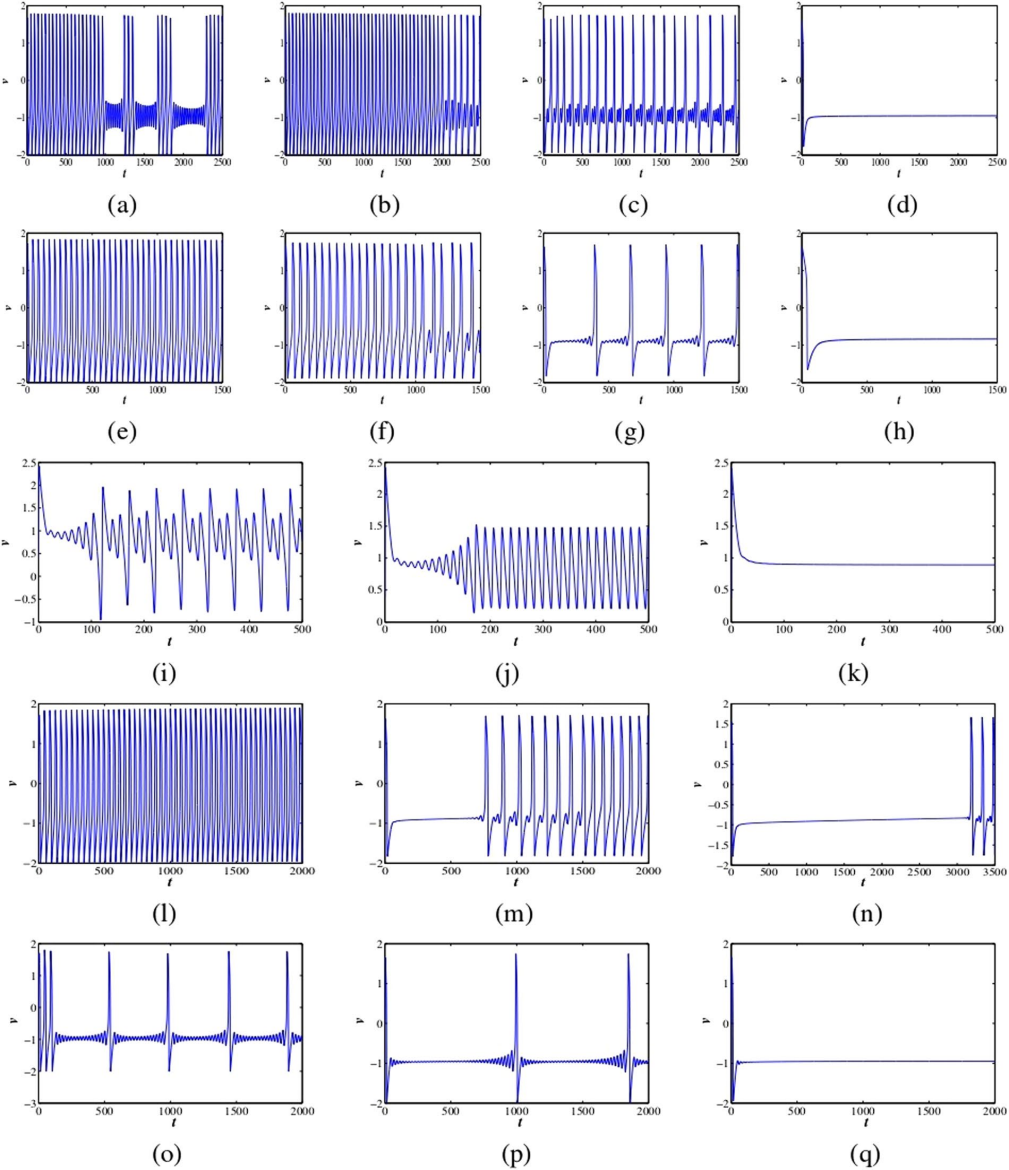
Different neuronal responses of membrane voltage (*v*) for the fractional-order FH-R model (1) at various fractional exponents, *α*. First panel: (a–d) $\alpha =1,0.98,0.95$ and 0.79 respectively (set I). Second panel: (e–h) $\alpha =1,0.92,0.85$ and 0.68, respectively (set II). Third panel: (i–k) $\alpha =1,0.99$ and 0.95 respectively (set III). Fourth panel: (l–n) $\alpha =1,0.85$ and 0.80 respectively (set IV). Fifth panel: (o–q) $\alpha =1,0.98$ and 0.95 respectively (set V).

Now, we study the fractional-order FH-R neuron model to investigate various firing activities. We evaluate how the classical order dynamics changes its neuronal behavior and how bursting changes to different firing patterns with respect to stability and bifurcation analysis for different fractional exponents. We used the time step $\Delta t=0.1$ for numerical results. We considered the initial conditions as small random perturbations around the fixed points for all the numerical simulations. Here the random perturbation is taken from a uniformly distributed random number in the interval (0, 1). An interesting feature is that the integer-order FH-R model produces elliptic bursting at the parameter set I. It generates decay and growth of small amplitude oscillations during the silent phase of bursting, and it is not damped rapidly. The firing pattern has multiple numbers of spikes in each burst having some subthreshold oscillations between two bursts (see Fig. 3(a)). The active and silent phases of bursting change as we slowly decrease the fractional exponent from classical/integer-order one $(0 < \alpha \le 1)$. At $\alpha =0.98$, it shows fast-spiking and after some time, it generates mixed-mode oscillations with high amplitude single spiking and low amplitude oscillations (see Fig. 3(b)). When it is further decreased to $\alpha =0.95$ (see Fig. 3(c)), it shows just mixed-mode oscillations. The fractional-order system has a Hopf bifurcation at $\alpha =0.80828$ and the system goes to quiescent state when $\alpha  < 0.80828$ i.e., it converges to the stable fixed point (${v}^{\ast }=-\,0.885098$) at $\alpha =0.79$ (see Fig. 3(d)). Now, we consider the parameter set II. The classical order system shows tonic spiking (see Fig. 3(e)). When the fractional exponent is decreased to $\alpha =0.92$, it displays a transition from tonic spiking into different spiking pattern with high amplitude and low amplitude oscillations (see Fig. 3(f)). The system shows mixed-mode oscillations at $\alpha =0.85$ (see Fig. 3(g)). There is first spike latency when the system is in this transition mode, and the firing frequency decreases. Then, the system exhibits a Hopf bifurcation at $\alpha =0.6951$, and it goes to complete quiescent state for $\alpha  < 0.6951$ shown in Fig. 3(h) (where ${v}^{\ast }=-\,0.841243$ and $\alpha =0.68$). Next, the classical order excitable model produces another spiking pattern with the parameter set III (see Fig. 3(i)). At $\alpha =0.99$, it shows regular low amplitude spikes relative to classical order model, and it has first spike latency (see Fig. 3(j)). Further, it converges to quiescent state at $\alpha =0.95$ (see Fig. 3(k)). Here, the system also converges to a stable fixed point (${v}^{\ast }=0.891229$). Next, we consider the parameter set IV. The classical order model shows fast-spiking (see Fig. 3(l)). Then, it produces first mixed-mode oscillations then regular spiking at $\alpha =0.85$ (see Fig. 3(m)). The system has first spike latency with the decrease of fractional-orders and firing frequency decreases. The first spike latency in the system increases with the decrease of fractional exponent $\alpha =0.80$ (see Fig. 3(n)). Finally, the parameter set V has been considered. The classical order model exhibits single high amplitude spikes with small amplitude oscillations not decaying to completely silent phase or oscillation death (see Fig. 3(o)). When the fractional-order is decreased, the spike frequency is decreased and the period of small amplitude oscillations increases, i.e., it is growing with larger time duration with $\alpha =0.98$ (see Fig. 3(p)). Finally, it goes to the complete quiescent phase at $\alpha =0.95$ i.e.; the system converges to the stable fixed point (${v}^{\ast }=-\,0.948702$) (see Fig. 3(q)).

**Figure 4:**
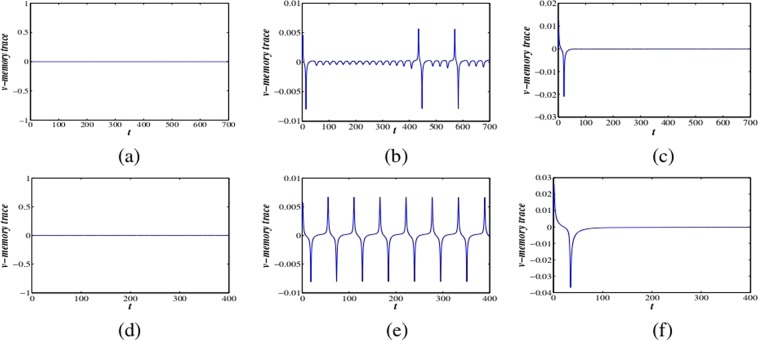
The dynamics of the voltage memory traces at fractional exponents (**a**–**c**) $\alpha =1,0.98,0.79$ and (**d**–**f**) $\alpha =1,0.90,0.69$ for parameter sets I and II respectively. For $\alpha =1$, the *v*-memory trace has no effect on the fractional dynamics. The nonlinearity in the system increases as we decrease the fractional-order (*α*) and the system shows no oscillation at lower values of *α*.

We characterize these diverse neuronal responses with stability and bifurcation analysis. The results show that the *v*-memory trace is zero at $\alpha =1$. The memory trace does not affect the dynamics of the system at $\alpha =1$ (see Fig. 4(a,d)). When fractional-order is decreased from classical order, new dynamical responses emerge. The voltage memory trace displays oscillations, i.e., it has major effects on membrane voltage dynamics and also membrane voltage affects the memory trace (see Fig. 4(b,e)). The fractional-order system is in steady-state at lower fractional-orders, *α* for all the parameter sets, i.e., the memory trace becomes too small, and it cannot significantly affect the membrane voltage dynamics to evoke a spike (see Fig. 4(c,f)). Therefore, the fractional-order excitable system changes to different oscillations as *α* increases above a threshold value for a fixed set of parameters.

#### Synchronization in coupled fractional FH-R models

**Figure 5:**
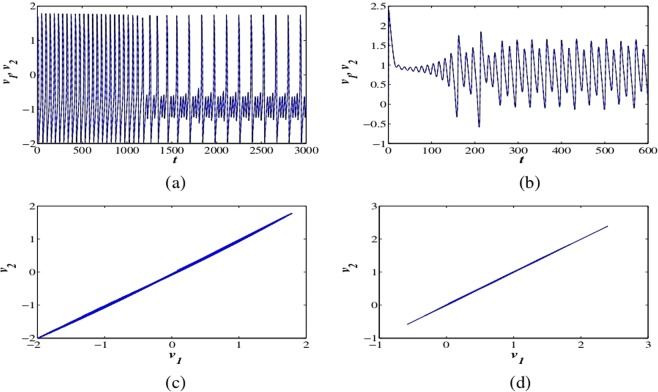
The membrane potentials of the excitatory coupled fractional-order FH-R model (at $\alpha =0.99$) for sets I and III with electrical coupling strengths (**a**) ${g}_{e}=0.55$ and (**b**) ${g}_{e}=0.3$ respectively. (**c**,**d**) *v*_1_ vs. *v*_2_ for the same fractional-order (*α*) and electrical coupling strength (*g*_*e*_). The membrane voltage variables *v*_1_ and *v*_2_ indicate strong correlation between them and exhibit CS.

**Figure 6:**
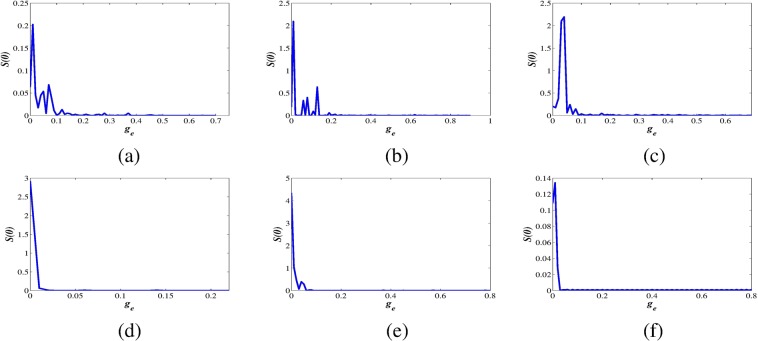
Similarity functions vs. coupling strengths for the fractional-order coupled FH-R system (8) with fractional exponents (**a**,**d**) $\alpha =1$, (**b**,**e**) $\alpha =0.99$ and (**c**,**f**) $\alpha =0.98$ at parameter sets I and III respectively. The synchronization error between the coupled neuronal oscillators converges to zero as we increase the coupling strength (*g*_*e*_) in a fixed range which confirms CS between the oscillators. However, with further decrease of *α*, it does not show CS.

To study the dynamics of the coupled FH-R neurons, we consider two synaptically coupled FH-R neurons in the fractional domain. Generally, synaptically coupled excitable cells produce in-phase or anti-phase synchronous activity depending on coupling structure and strengths. We show the transitions to complete synchronization^37,61,63^ (CS) regime of the coupled fractional-order excitable neurons. We use bidirectional coupling, i.e., electrical coupling between two FH-R neurons, which is biophysically efficient synaptic coupling mechanism.

8 $$\begin{array}{ccc}\frac{{d}^{\alpha }{v}_{i}}{d{t}^{\alpha }} & = & {v}_{i}-{v}_{i}^{3}/3-{w}_{i}+{y}_{i}+I+{g}_{e}({v}_{j}-{v}_{i}),\\ \frac{{d}^{\alpha }{w}_{i}}{d{t}^{\alpha }} & = & \delta (a+{v}_{i}-b{w}_{i}),\\ \frac{{d}^{\alpha }{y}_{i}}{d{t}^{\alpha }} & = & \mu (c-{v}_{i}-d{y}_{i}),\end{array}$$

where *g*_*e*_ is the coupling strength, $i=1,2$ and $j=2,1$. The above system is bidirectionally coupled via membrane voltage. The synchronization regimes and its stability are examined by similarity functions^64^. Complete synchronization of these two coupled systems indicates the stability of zero solutions of the error system. The desired synchronization state is achieved by using suitable coupling strengths and appropriate fractional exponents at different parameter sets (see Fig. 5). Now, we introduce a statistical measure known as similarity function to estimate the synchronization error between the coupled neuronal oscillators to produce CS. The function is defined as ${S}^{2}(\gamma )=\langle {({v}_{1}(t)-{v}_{2}(t-\gamma ))}^{2}\rangle /{(\langle {v}_{1}^{2}(t)\rangle \langle {v}_{2}^{2}(t)\rangle )}^{1/2}$ and *S*(*γ*) measures the phase lag between the coupled excitable systems. The smaller value of *S*(0) shows a high correlation between driver and response oscillators. The functional value *S*(0) confirms CS (see Fig. 6) regime as it converges to zero with different initial conditions. This verified the coupling scheme and effectiveness of the method for synchronization.

## Discussion

Fractional-order dynamics has more advantages to real-world applications. It may produce complex dynamics, such as switching to different stabilities, periodic nature, and chaotic behavior. Our nonlinear fractional-order biophysical model shows such types of complex dynamics. The theoretical analysis and numerical results reveal some interesting neuronal responses that can be useful for further investigation of the fractional-order excitable systems. The characteristics of a fast-slow FH-R model has been introduced in this article by using a commensurate fractional-order derivative. It has been examined that how fractional exponents influence the dynamics of the system and make it different from classical-order FH-R model that exhibits elliptic bursting. It shows different types of oscillations; spike frequencies based on different sets of parameters. The fractional exponent plays a significant role in generating and destroying bursting. It changes the nature of the system dynamics. It also makes us to understand the information processing in coupled systems^11,20,37,39^. Synchronization of fractional-order excitable systems, especially chaotic systems has potential applications to control secure communication^63^. We observed that spikes also produce for small fractional-orders in the fast-slow neuron model. The transition states for various firing modes, including the quiescent states are discussed for different fractional-orders with various sets of parameters. The significance of our work is that we consider biologically relevant electrical coupling, i.e., bidirectional coupling for two neurons showing different types of oscillations and establish the CS criterion in fractional-order coupled systems. It can be extended to a network of neurons with such type of fractional-order neurons for a bidirectional or gap junction coupling scheme^11,42^. It has become a challenging task to select suitable neuron model with appropriate parameter sets that exhibits different dynamical behavior when we introduce the fractional-order component in the system. This type of study of excitable biophysical systems is limited as different complex mathematical solutions arise; however, some techniques have already been developed to investigate the fractional-order dynamics. It may play a significant role in understanding the signal processing dynamics, noise-induced electrical activity, the synaptic mechanism in neuronal populations, different neurocomputational features, properties of different types of neural networks for complex brain functioning in healthy and diseased state conditions such as neurological disorders. Further study with fractional-order dynamics is needed to investigate the excitable single neuron model with its coupled nature and the dynamics of the different structured excitatory-inhibitory neuronal population.
